# Age-related reduction of adaptive brain response during semantic integration is associated with gray matter reduction

**DOI:** 10.1371/journal.pone.0189462

**Published:** 2017-12-13

**Authors:** Zude Zhu, Fengjun Yang, Dongning Li, Lianjun Zhou, Ying Liu, Ying Zhang, Xuezhi Chen

**Affiliations:** 1 School of Linguistics Sciences and Arts, and Collaborative Innovation Center for Language Competence, Jiangsu Normal University, Xuzhou, China; 2 Ningcheng Central Hospital, Chifeng, China; 3 Sucheng People’s Hospital, Suqian, China; Hangzhou Normal University, CHINA

## Abstract

While aging is associated with increased knowledge, it is also associated with decreased semantic integration. To investigate brain activation changes during semantic integration, a sample of forty-eight 25–75 year-old adults read sentences with high cloze (HC) and low cloze (LC) probability while functional magnetic resonance imaging was conducted. Significant age-related reduction of cloze effect (LC vs. HC) was found in several regions, especially the left middle frontal gyrus (MFG) and right inferior frontal gyrus (IFG), which play an important role in semantic integration. Moreover, when accounting for global gray matter volume reduction, the age-cloze correlation in the left MFG and right IFG was absent. The results suggest that brain structural atrophy may disrupt brain response in aging brains, which then show less brain engagement in semantic integration.

## Introduction

Language comprehension is a critical ability for daily life. Semantic integration is a key process in understanding meaning from any given flow of information. Differing from word recognition and retrieval, semantic integration combines small pieces of word-level information into larger message-level representations [1]. While aging is associated with increased vocabulary and augmented stores of world knowledge [2], cumulative evidence has revealed age-related declines in semantic integration during sentence comprehension [3,4].

Event-related potential (ERP) studies have shown that in younger adults, semantic integration is associated with an N400 effect [5,6], with more negative deflection occurring when semantic integration difficulty increases. Semantic integration is also associated with brain activation in multiple brain regions, including the bilateral inferior/middle frontal gyrus (I/MFG), the bilateral middle temporal gyrus (MTG), and the anterior temporal lobe (ATL). This activation has been demonstrated in functional magnetic resonance imaging (fMRI) studies [1,5,7–10].

ERP studies have revealed a much smaller N400 effect in older adults than in younger adults [3,11], with delayed onset of the N400 effect having been reported [12]. What is more, the N400 effect appears to decline linearly with age in older adults. For instance, one study showed that among older adults in their fifties, sixties, and seventies, the smallest N400 effect occurred in people in their seventies [13]. Taking advantage of high temporal resolution, ERP studies have thus provided clear evidence of age-related decline in semantic integration ability. However, the relationship between semantic integration decline and brain activation change in aging still remains unclear. While fMRI studies have focused mainly on semantic processing at the word level [14–19] and on syntactic processing [20–23], a handful of studies have investigated brain function changes in semantic integration during sentence comprehension [22,24].

Erb and Oblesor (2013) presented both degraded and clear auditory sentences to younger and older adults. While younger and older adults showed largely overlapping activation during degraded speech processing, comprehension scores were positively correlated with brain activity in the right MFG in older adults only, and in the left fusiform gyrus, cerebellum, and posterior cingulate cortex in younger adults only. The degrade effect (degraded versus clear speech) was negatively correlated with hearing loss in the bilateral insula. This manifested as an increased blood-oxygen-level dependent (BOLD) signal for clear relative to degraded speech being associated with greater hearing loss. The comprehension score was positively correlated with brain activity in the anterior cingulate cortex (ACC) in both groups, with older adults showing worse performance and lower activity. However, the results revealed that comprehension of degraded speech greatly relied on cognitive control, for which there is a declined capacity in older adults [2,25].

One more study [26] relevant to semantic integration compared brain activation of seniors with lower versus higher accuracy during grammatically simple sentence reading. Seniors with poorer comprehension showed higher activation in the bilateral I/MFG and in the ACC than those with better comprehension. However, the group comparison in this study was based on the entire simple sentence (relative to the baseline), which could not separate semantic integration from syntax processing. Both age-related change and preservation have been reported when syntax complexity increases during speech comprehension [20,21,27,28]. Again, syntax-complexity-induced comprehension difficulty is beyond difficulty in semantic integration per se.

Therefore it is necessary to identify the age-related brain function changes that are responsible for semantic integration, rather than other cognitive processes that may accompany semantic integration. To this end, we constructed high cloze (HC) and low cloze (LC) sentences [29]. High-cloze sentences were sentences in which a noun at a given position (the critical word) was highly expected. In contrast, low-cloze sentences were semantically correct, but the noun at the critical word position was unexpected. Semantic integration appears to be more difficult in LC than in HC sentences, which is evident from larger N400 amplitudes [1,5]. However, compared with reading congruent sentences, reading violation sentences not only increases the integration difficulty but also involves other cognitive processes such as error monitoring and repairing [30,31]. To avoid tapping processes other than semantic integration, the present study focused on the differences between LC and HC sentences (the cloze effect). By employing a sample with an age range from 25 to 75 years old, age-related changes associated with LC versus HC differences were investigated. Since aging has been associated with a smaller N400 effect, it is possible that a smaller cloze effect would be manifested in regions that contribute to semantic integration, including left prefrontal cortex and left temporal cortex.

Moreover, cumulative evidence has demonstrated age-related cerebral gray matter shrinkage [32,33]. Recent studies have shown that reduced gray matter also contributes to language decline [19,34,35]. An interesting question is whether age-related functional alteration could also be explained by gray matter reduction, or if age itself is rather the main cause of brain activity change. According to the mediation hypothesis [36], the association between age and brain function change would disappear if the gray matter reduction fully mediated the age-related function decline; otherwise the association would persist if the gray matter reduction is independent of the age-related function decline [37].

## Methods

### Participants

A total of 48 right-handed, monolingual Chinese speakers (28 males) participated in this study. Mean age was 49.7 years old (SD 15.3, range 25–75). The number of people in their twenties, thirties, forties, fifties, sixties, and seventies was 8, 8, 6, 13, 8, and 5, respectively, with no significant gender ratio difference across these age subgroups, *X*^2^ = 8.86, *df* = 5, *p* = 0.15). Mean years of education was 11.4 (SD 2.8, range 6–16). The study was approved by the Ningcheng Central Hospital review board, according to the Declaration of Helsinki. Written informed consent was obtained from each participant immediately prior to the study. Participants were residents of the community who had normal or corrected-to-normal visual acuity. Exclusionary criteria for the study included the following: a major head injury, stroke, a neurological or psychiatric disorder, high blood pressure, diabetes, heart disease, the use of psychotropic drugs, or the presence of metal fragments and/or metallic implants contraindicated for MRIs. Before the MRI, the Beijing version of the Montreal Cognitive Assessment (MoCA, http://www.mocatest.org/) was administered to test short-term memory recall, visuospatial skill, executive functioning, language, orientation, attention, concentration, and working memory. The mean score on the MoCA was 27.2 (SD 1.7, range 25–30), indicating that the participants were cognitively healthy.

### Stimuli

To manipulate semantic prediction, we used high cloze (HC) and low cloze (LC) sentences that were modified from a previous study [1]. First, we constructed 144 sentences (each 8 to 13 words in length) with highly constraining contexts. The critical word (CW) appeared at the end of the Chinese sentence (e.g., 放学的小明正背着*书包*/After school, Peter was carrying his *backpack*). The CW was then replaced with a semantically unexpected noun (足球/football) that was nevertheless semantically congruent within the context of the LC condition (Table 1). The cloze probability was rated by another 58 participants from the same participant pool. The cloze probabilities of the HC condition (the ratio of the most frequently appearing word in all self-generated responses) ranged from 59% to 100% (mean ± SD, 89.5 ± 10.7%). The cloze probabilities of the high cloze condition were significantly higher than the LC condition (1.0 ± 1.8%, ranging from 0 to 8.6%, *t* (143) = 91.7, *p* < 0.001). The CWs were matched across conditions for frequency (log frequency for HC and LC were 2.4 ± 0.7 and 2.4 ± 0.6, respectively, *p* > 0.5) [38] and the number of character strokes controlling for visual complexity (HC and LC were 16.3 ± 4.5 and 15.9 ± 4.3, respectively, *p* > 0.5), which is akin to word length in English. Semantic acceptability for all sentences was rated on a 6-point Likert scale (1 = entirely unacceptable, 6 = fully acceptable) by 18 participants from the same pool. The average ratings for the HC and LC conditions were 5.3 ± 0.4 and 5.2 ± 0.4. The acceptability ratings were comparable between HC and LC conditions (*t* (143) = 1.27, *p* = 0.21). Sentence presentation was counterbalanced across participants.

**Table 1:** Stimuli examples and control data (mean ± SD).

Conditions	Sentences	Frequency	Number of Strokes	Semantic Acceptability
High cloze (HC)	刚放学的小明背着书包。 After school, Peter was carrying his backpack.	2.4 ± 0.7	16.2 ± 4.3	5.3 ± 0.4
Low cloze (LC)	刚放学的小明背着足球。 After school, Peter was carrying his football.	2.4 ± 0.6	15.9 ± 4.4	5.2 ± 0.4

Note. Critical words were underlined.

### Procedure

All stimuli were presented using E-Prime (version 1.1) software. The sentences were presented word by word, with words displayed for 500 ms, followed by a 100 ms blank. There was a 200 ms fixation screen prior to presentation of the first word. The words were presented in Song font with a font size of 40, and the words were black against a gray background. After the sentence presentation, participants were asked to press a button within a 4000 ms time window to indicate whether the sentence was semantically acceptable. The durations of the inter-trial intervals were randomly selected between 200–6200 ms. The sentences were presented in two runs.

### fMRI data acquisition

Data acquisition was performed using a Philips 1.5 T MR scanner. Whole-brain echo-planar images (EPIs) were acquired in an interleaved manner with ascending slice order (TR = 2000 ms, TE = 30 ms, flip angle = 77°, 40 slices, voxel size = 3.5 × 3.5 × 3.5 mm^3^). A high-resolution T1-weighted scan was acquired for each participant after the functional runs using an MPRAGE sequence (192 slices, TE = 2.93 ms; slice thickness = 1mm; voxel size = 1 × 0.875 × 1 mm^3^).

### fMRI preprocessing

The fMRI Expert Analysis Tool (FEAT) v5.0.8, part of the FMRIB Software Library (FSL) was used in the preprocessing and statistical analyses of the fMRI data. Data preprocessing steps were similar to those used in our previous work [39]. Data were first motion corrected, smoothed with an 8 mm Gaussian kernel, and highpass filtered at 100 s. fMRI data were then co-registered to each individual’s high resolution structural scan using boundary-based registration. The high resolution structural image was co-registered to MNI 2 × 2 × 2 mm^3^ space using an initial linear registration followed by nonlinear warping (using FNIRT). These transformation parameters were then applied to the functional data, which was re-sliced to 2 mm isotropic voxels during non-linear warping into MNI space.

Functional data was first modelled at the individual subject level by fitting a voxel-wise General Linear Model (GLM) to the BOLD data acquired from each run. Task regressors for HC and LC, and wrong trials across conditions, were modelled as a box-car function and convolved with a canonical double gamma hemodynamic response function for each run. Using a fixed-effects model, in the 2^nd^ level model, maps for each condition were averaged across the two runs within each participant.

### fMRI statistical analyses

Group analyses focused on the cloze effect (LC-HC). Monte Carlo simulations using the AlphaSim program were used to determine the appropriate combination of significance level and cluster threshold required to reach a corrected significance level of *p* < 0.05. This took into account both native space voxel dimensions and the effective smoothness estimated directly from our preprocessed data (http://www.restfmri.net). The Monte Carlo simulations used 1000 iterations and indicated a significance level of *p* < 0.005 and a cluster threshold of 60 voxels in order to reach a corrected significance level of *p* < 0.05. This threshold was applied to each of the contrasts described in the study.

### Voxel-based morphometric analyses

To expose age-related gray matter reduction, each participant’s T1 image was used to perform voxel-based morphometric (VBM) analysis according to the procedure in FSL-VBM [40]. The structural images were brain-extracted and gray matter was segmented before being registered to the MNI 152 standard space using non-linear registration. The resulting images were averaged and flipped along the x-axis to create a left-right symmetric, study-specific gray matter template. All native gray matter images were then non-linearly registered to this study-specific template and "modulated" to correct for local expansion (or contraction) due to the non-linear component of the spatial transformation. Finally, the modulated gray matter images were smoothed with an isotropic Gaussian kernel with a sigma of 3 mm. After preprocessing, a GLM analysis was conducted using age as a predictor, gender and education as covariates, and gray matter reduction as the dependent variable.

### Voxel-wise age-cloze correlation

In order to reveal age-related changes in the cloze effect, the cloze effect contrast (LC-HC) of each subject underwent a covariate analysis at the group level, with age as a predictor and education and gender as covariates. To further explore whether age-related brain function changes associated with the cloze effect were influenced by brain volume reduction, a voxel-wise general linear model analysis was conducted. Age and global regional volume reduction were used as regressors, and brain function as the dependent variable. Education and gender were used as covariates, but they were not of primary interest.

### ROI analysis

Because previous studies suggested that the left prefrontal cortex (PFC) plays an important role in sentence-level semantic integration, a region of interest (ROI) analysis was also conducted to test the relationship between age and the cloze effect in the left PFC. ROIs in the left middle frontal gyrus (MFG, MNI coordinates: -40 24 24) and the inferior frontal gyrus (IFG, MNI coordinates: -38 40–10) were utilized from our previous study [1], which demonstrated the cloze effect in both explicit and implicit semantic tasks. The significant correlation identified in previous step was then further tested after the VBM was added into the regression model.

## Results

### Behavioral results

Behavioral results revealed a significant cloze effect in terms of both accuracy and response time (RTs). The participants comprehended the HC sentences with high accuracy (92.6 ± 8.0%); however, they performed significantly worse on the LC sentences (68.5 ± 12.0%), *F* (1, 47) = 180.4, *p* < 0.001. The RTs in the LC condition (1382 ± 290 ms) were also significantly slower than those in the HC condition (1716 ± 393 ms), *F* (1, 47) = 109.4, *p* < 0.001.

The cloze effect for accuracy (*r* = -0.10, *p* = 0.52) and RTs (*r* = -0.06, *p* = 0.68) were not significantly correlated with age. However, the accuracy increase in HC (*r* = -0.32, *p* = 0.027) and LC (*r* = -0.28, *p* = 0.05) was negatively correlated with age. The RTs increase in HC (*r* = 0.47, *p* = 0.001) and LC (*r* = 0.29, *p* = 0.046) was positively correlated with age. The results indicated that within each condition, an increase in age was associated with worse performance.

### fMRI results

**Fig 1:**
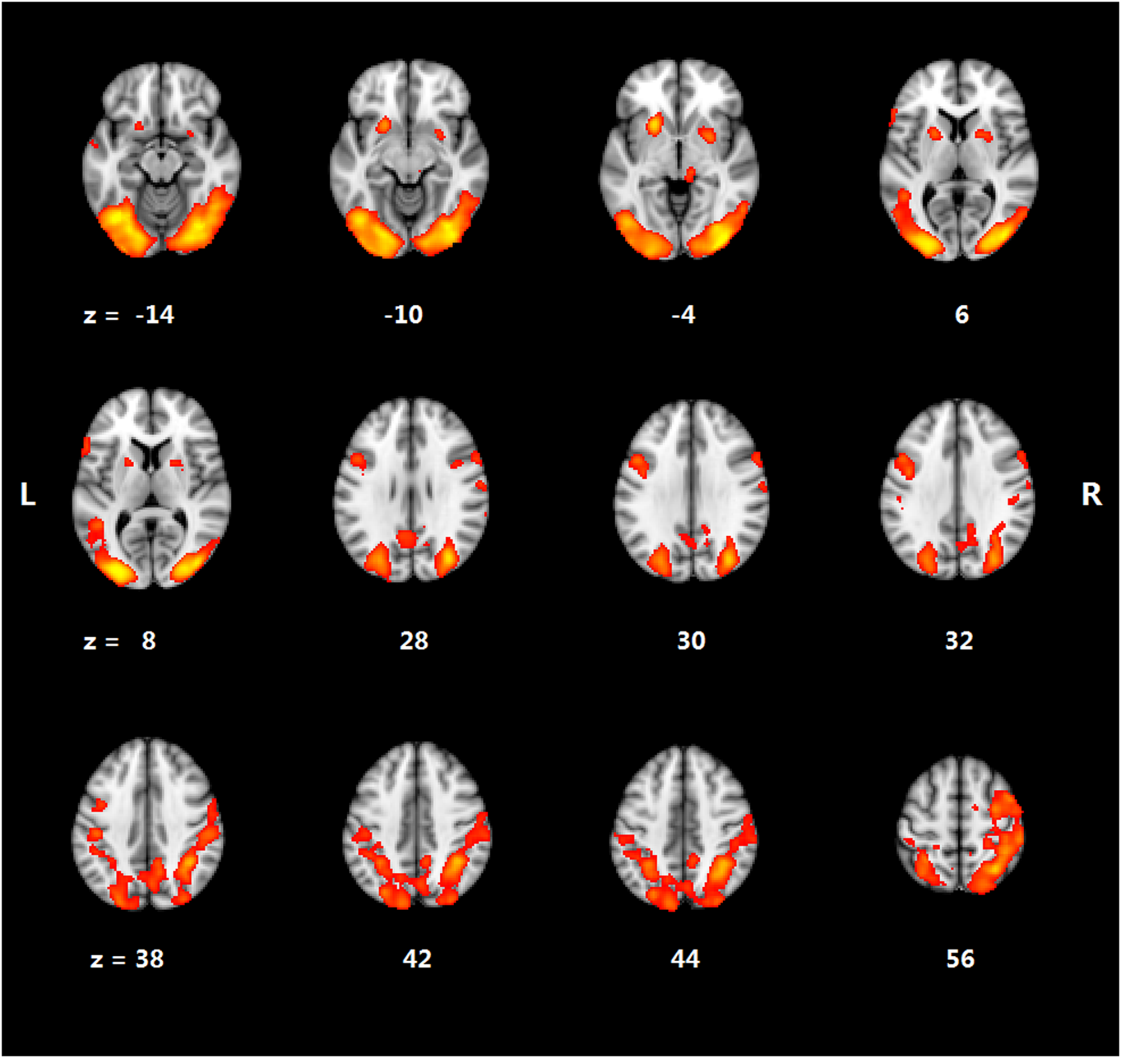
Brain activation for cloze effect.

Significant activation occurred in the frontal, temporal, and parietal regions in both the HC and LC conditions. For the cloze effect, Table 2 and Fig 1 depict significantly higher activation in the LC than in the HC condition in several regions. This includes the left middle frontal gyrus (MFG), the left inferior frontal gyrus (IFG), the left anterior temporal lobe (ATL), the right supramarginal gyrus (SMG), the right thalamus, the bilateral occipital/temporal cortex, and the bilateral putamen.

**Table 2:** Cloze effect on brain activation.

Region	Hem	Voxels	Z-score	X	Y	Z
LC > HC						
Middle frontal gyrus/Precentralgyrus	Left	361	3.70	-42	0	34
Inferior frontal gyrus	Left	95	3.21	-56	20	10
Occipital/Temporal cortex/Superior parietal lobule	Bilateral	6235	5.51	-30	-88	14
Anterior temporal lobe	Left	178	4.06	-56	8	-24
SupramarginalGyrus	Right	73	3.13	62	-38	20
Putamen	Left	516	4.70	-22	14	-4
Putamen	Right	373	3.79	28	4	-2
Thalamus	Right	92	3.28	12	-28	-4
HC > LC	N.S.					

Note. LC: low cloze sentences; HC: high cloze sentences; Hem: hemisphere. X, Y and Z indicate coordinates in MNI standard space. N.S., no significant difference.

### Age-VBM correlation

**Fig 2:**
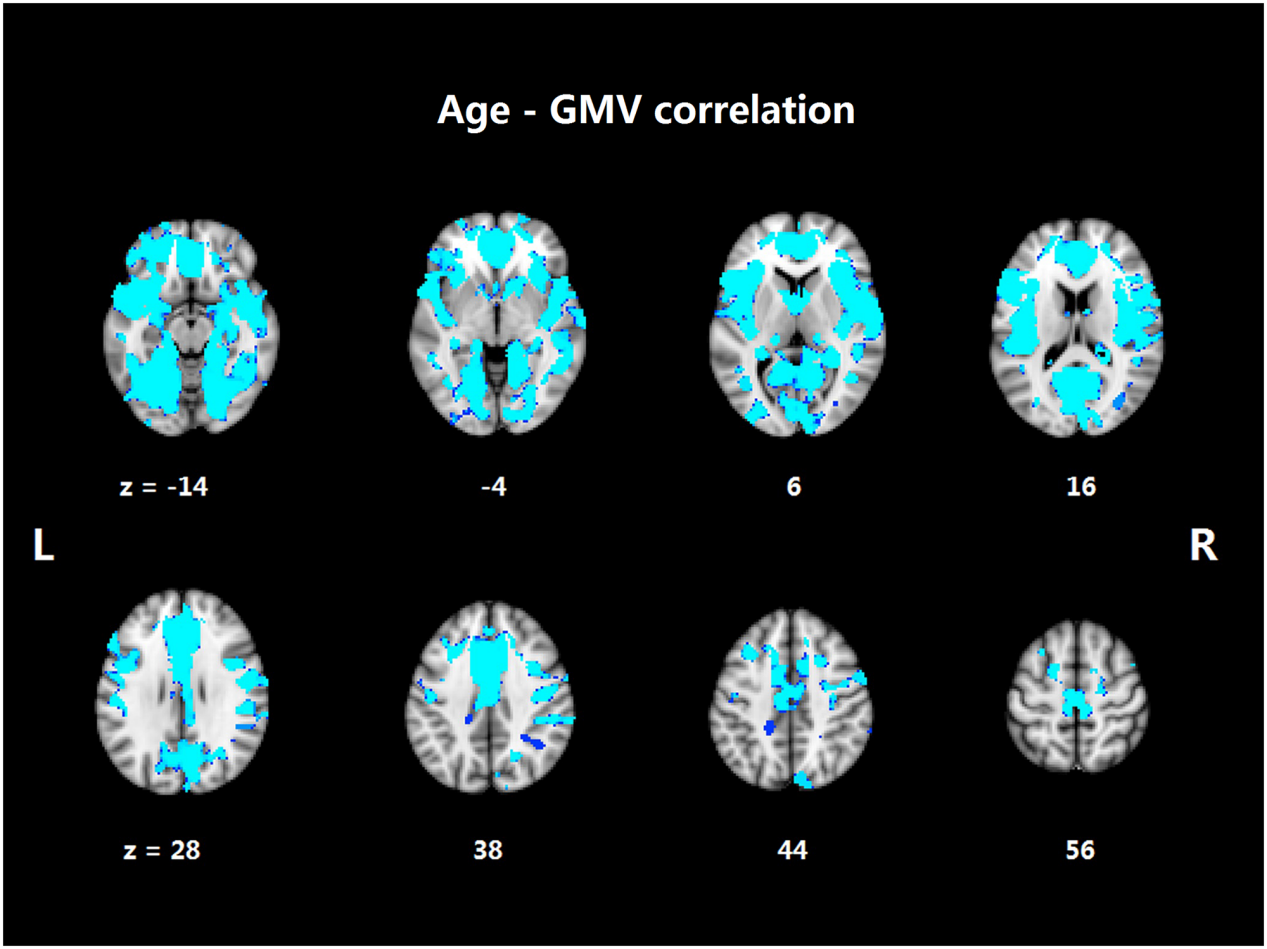
Aging-associated gray matter volume (GMV) reduction.

Fig 2 shows the significant negative correlation between age and gray matter volume in widespread regions across the whole brain. These regions included the bilateral IFG and MFG, the bilateral superior/middle/inferior temporal gyrus (S/M/ITG), the bilateral hippocampus, the bilateral occipital and parietal lobe, the cingulate cortex, the bilateral insula, and the basal ganglia.

### Age-cloze correlation

**Fig 3:**
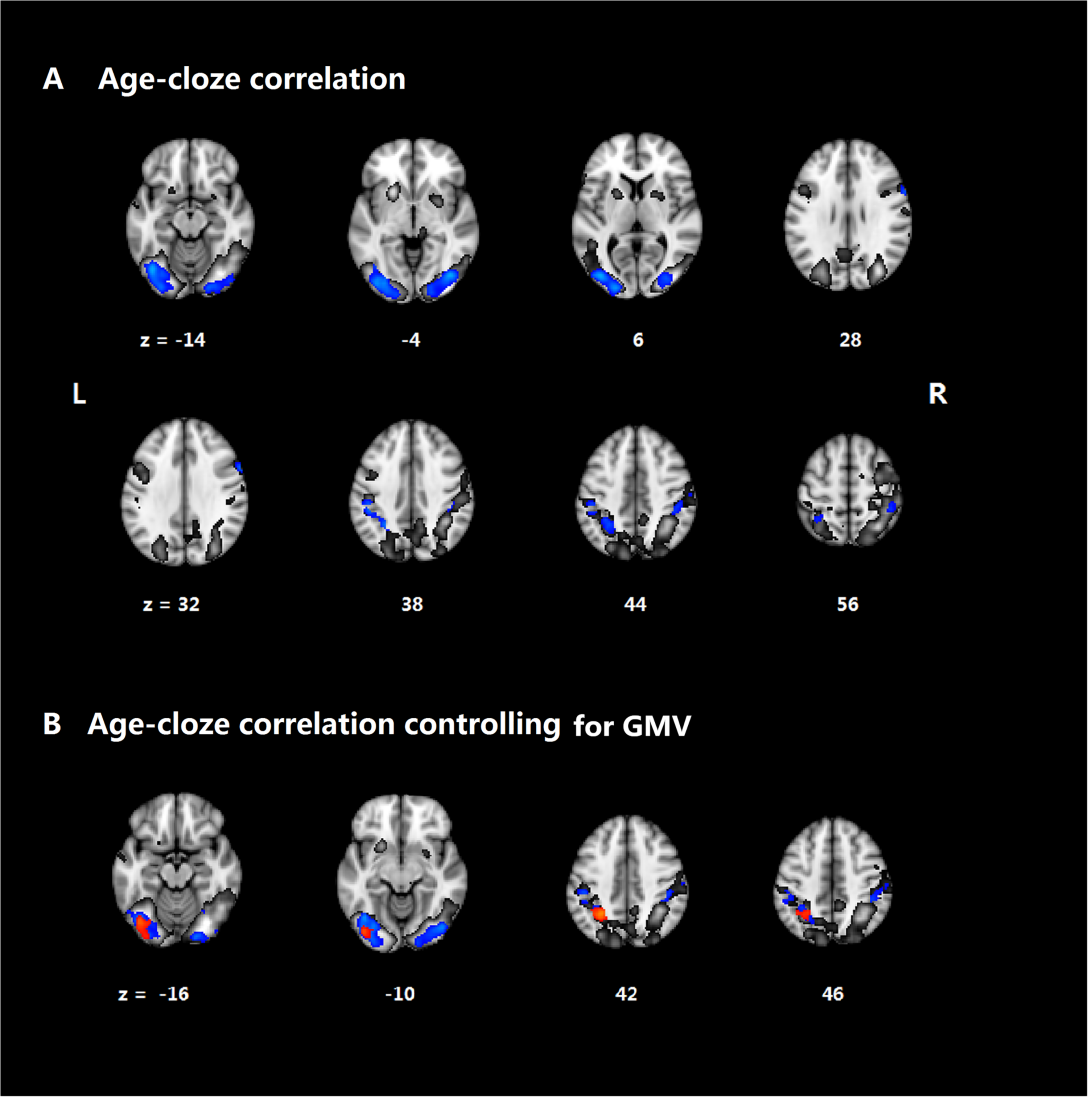
Aging-cloze correlation. (A) Aging-related cloze effect controlling for gender and education (blue), within the cloze effect regions (gray). (B) Aging-related cloze effect controlling for gender, education and gray matter volume (GMV) (red) within the cloze effect regions (gray) and age-cloze effect presented in (A) (blue).

The cloze effect seen in BOLD was significantly reduced in age. As shown in Table 3 and Fig 3A, significant negative BOLD-age correlations were found for the right IFG, the bilateral inferior occipital cortex (IOC), the bilateral SMG, and the right cerebellum. No significant positive correlations were found.

**Table 3:** Regions showing significant age-cloze effect correlation.

Region	Hem	Voxels	Z-score	X	Y	Z
*Negative correlation*						
Inferior frontal gyrus/Precentralgyrus	Right	73	3.44	60	12	32
Inferior occipital gyrus	Left	1372	4.24	-22	-94	2
Inferior occipital gyrus	Right	1352	4.27	42	-76	-6
Superior parietal lobule/supramarginalgyrus	Left	526	3.72	-28	-52	40
Supramarginalgyrus	Right	297	3.48	44	-38	50
Cerebellum	Right	117	3.39	32	-64	-24
Cerebellum	Right	108	3.16	44	-44	-32
Cerebellum	Right	80	3.48	52	-64	-24
*Positive correlation*	N.S.					

Note. Hem: hemisphere. X, Y and Z indicate coordinates in MNI standard space. N.S., no significant difference.

Controlling for gray matter reduction, the significant correlation between age and cloze effect was still observed in the left IOC, the left SMG and the right cerebellum, but not in the right IFG, right IOC, or right SMG. This is shown in Fig 3B and Table 4.

**Table 4:** Significant age-cloze effect correlations after controlling for gray matter volume.

Region	Hem	Voxels	Z-score	X	Y	Z
*Negative correlation*						
Inferior occipital cortex	Left	374	3.44	-40	-76	-14
Superior Parietal Lobule	Left	199	4.08	-28	-52	38
Cerebellum	Right	68	2.95	32	-74	-22
*Positive correlation*	N.S.					

Note. Hem: hemisphere. X, Y and Z indicate coordinates in MNI standard space. N.S., no significant difference.

For the ROI analysis, age was significantly correlated with the cloze effect in the left MFG (*r* = -0.26, *p* < 0.05) but not in the left IFG (*r* = 0.01, *p* = 0.96). For the left MFG, the age-cloze effect correlation was not significant after controlling for global volume reduction (*t* = -0.38, *p* = 0.71).

## Discussion

Two main findings were discovered in the current study. First, age-related functional and structural alteration was found. Age-related volume reduction occurred in widespread regions, and a smaller cloze effect occurred in the frontal, parietal, and temporal regions. Second, the age-cloze correlation was significant in the left IOC and SMG, but not in the left MFG, right IOC, or right SMG, after controlling for whole brain volume reduction. These results shed light on our understanding of the neural underpinnings of age-related changes during comprehension.

The cloze effect has been frequently implicated in the examination of semantic integration during sentence comprehension. In the present study, in line with previous studies [4,13], there was a significant cloze effect across younger and older adults but no significant age-cloze effect correlation for accuracy or RTs. In contrast, increases in age were associated with lower accuracy and longer RTs. The RT-age correlation suggests that reduced processing speed may contribute to the reading comprehension aging effect [41]. However, as accuracy was also worse in the older adults than younger adults, the result suggests more difficulty in reading comprehension for older adults than younger adults. Previous studies found that the cloze effect related to BOLD activation increases in frontal and temporal regions in young adults [1,42,43]. In line with the literature, the present study revealed higher activation in the LC condition than in the HC condition, for the frontal, temporal-occipital, and parietal regions.

Although we mainly relied on ERPs to investigate age-related changes during semantic integration, the present age-cloze effect correlation in brain activity provides evidence helpful to understanding the brain regions associated with semantic integration in aging. Specifically, the fMRI results showed a clear age modulation of the cloze effect. That is, older adults showed a smaller BOLD cloze effect in the bilateral IOC, SMG, and right IFG in voxel-wise analysis, and in the left MFG in ROI analysis. These results align well with previous ERP studies that have revealed a smaller N400 effect in older adults than younger adults during sentence comprehension [4,44,45]. Previous studies have demonstrated that the bilateral prefrontal cortex, including the left MFG and right IFG reported on here, play an important role in sentence-level semantic integration. However, verbal working memory has been associated with the SMG [46]. The correlation between age and cloze effect thus suggests that aging is not only involved in a decline in semantic integration per se, but also in a decline in supportive processes such as verbal working memory and visual processing.

Under-recruitment in older adults is common during receptive linguistic processing. Pertaining to this, the results found in the present study are consistent with other research. For instance, under-recruitment in the left frontal-temporal regions was found more in poor older readers than in good older readers [22]. It was also found in older adults more than in younger adults during grammatically complex sentence reading [23] and speech perception [19]. Similarly, previous studies have also found age-related under-recruitment in lexical semantic processing [17], word recognition [47], and semantic coding [48]. Under-recruitment may reflect not enough resources [2] in older adults engaged in high demand tasks. Due to the fact that the critical words in the present study did not match the context, the LC sentences may not only have demanded more semantic integration processing in the frontal cortex, but may also have taxed verbal working memory in the SMG [46]. LC sentences might also have elicited more visual processing in the bilateral IOC than the HC sentences. However, older adults may not be able to effectively recruit the integration and supportive processes in comprehension and thus under-recruit brain activations.

Such under-recruitment may also be due to structural decline. Our study, consistent with the literature [33,35,49], found that smaller gray matter volume in older adults was widespread in the cortical and subcortical cortex. Although the age-related volume reduction did not perfectly coincide with the age-related cloze effect, volume reduction was clearly observed in brain regions that are involved in sentence comprehension. These areas include the bilateral frontal-temporal cortex and the temporal-occipital cortex. Because previous studies found a high consistency in global age-related decline, but not in single region atrophy, and the present study found a single widespread cluster after multiple comparison correction, the mean value from the whole volume reduction mask was used in further analysis. When the whole brain volume reduction was added to the regression model, the age-related cloze effect was significantly different than it was without the volume reduction. Specifically, the age-cloze effect correlation in the left IOC and left SMG was still significant. However, that correlation in the left MFG, right IFG, right IOC and right SMG was not significant after the whole brain volume reduction was added in the model.

Thus, the results here suggest that whole brain volume reduction did in fact explain age-related brain functional changes during sentence comprehension. The smaller cloze effect for older adults than younger adults in the specified regions suggests that the brain is less likely to be modulated during semantic integration in aging. The reduction may be due to the globally decreased gray matter. Such a function-structural correlation underscores the findings of recent studies showing that decreased gray matter volume is associated with decreased connectivity within key language regions [19,35]. The age-cloze effect and structural correlations may also explain the previous findings in ERPs. Smaller N400 effects have been demonstrated more in older adults than in younger adults, suggesting that these effects in older adults are less likely to predict upcoming words during language comprehension [3]. Since the previously absent age-cloze correlation was found in the present study in core language regions including the left MFG and right IFG, the results imply that less utilizing prediction by older adults may be due to their having fewer adaptive responses than younger adults.

The left IOC and SMG however, still showed an age-cloze effect with the brain cluster extension more restricted, after volume was controlled for. The IOC and SMG were not part of expected regions for semantic integration. The activation in left IOC during sentence comprehension [31] was associated with reading related visual processing, and the activation in left SMG was associated with working memory demand [50], not semantic integration per se [1,5]. So while the regions could exhibit structural-function correlations [51] in other cognitive functions, they may not necessarily do so in sentence comprehension. Together with the results showing that gray matter reduction explained the age-cloze correlation, these findings suggest heterogeneity of the brain regions in the relationship between age, brain function, and structural change.

One limitation of the present study is that structural changes should be interpreted in the context of gray matter volume reduction. Beyond gray matter volume, structural alteration can also be measured by cortical thickness for gray matter and diffusion tensor imaging [47] and kurtosis imaging [52] for white matter. The latter indices have contributed to understanding functional changes in aging brains, and further investigation is needed to elucidate their specific nature. Moreover, it has been found that older and younger adults might be different in using context constraints during comprehension. Future studies should try to manipulate context constraints to reveal the brain functional and structural basis for sentence comprehension in aging.

In summary, the present study examined brain activity patterns in aging during the comprehension of sentences with varied semantic integration difficulty. The results revealed an age-related decline in brain adaptive changes for integration difficulty. Part of such a decline can be explained by brain volume reduction, suggesting that disrupted brain structures may induce less brain functioning.

## Supporting information

S1 Table Relevant demographic, behavioral and VBM data underlying the findings described in manuscript. ID, subject ID used in the present study; Age, years of age; Gender, 1 for male and 2 for female; MoCA, score for MoCA test; Yr_edu: years of education; Acc_HC, accuracy for High Colze condition; Acc_LC, accuracy for Low Cloze condition; RT_HC, response time for High Colze condition; RT_LC, response time for Low Cloze condition; VBM, mean of VBM in the whole brain masked regions.
(XLSX)
